# 

**Published:** 1990-03

**Authors:** 


V.105 NO.1 1990

C.01---------SEQ: SR0068788

TI: WEST OF ENGLAND MEDICAL

JOURNAL

### WEST OF ENGLAND MEDICAL JOURNAL

Volume 105(i) March 1990

Some Plymouth Worthies
Coronaries, Cholesterol and Children
Diet, Dermatology and Rotten Fish
Prevention of Down's Syndrome
Fats and Cancer
Carcinoma of the Pancreas
Breast Cancer Screening
A New Approach to Medical Records
Thyroxine and Osteoporosis
Monetarism and General Practice

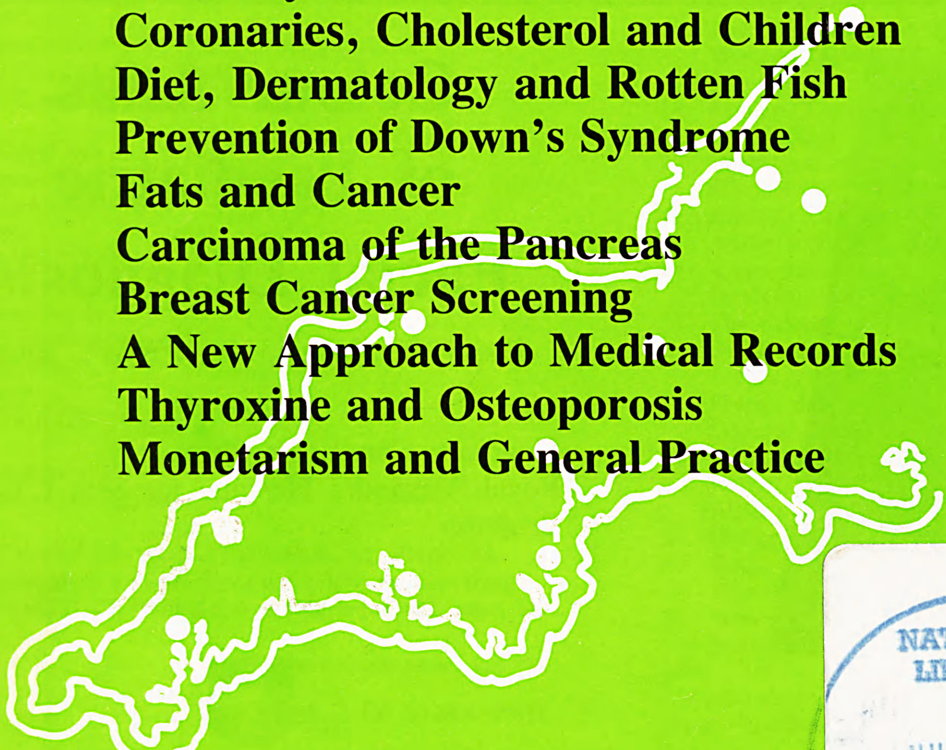

NATIONAL LIBRARY
JUL 16 1990
OF MEDICINE

FORMERLY BRISTOL MEDICO-CHIRURGICAL JOURNAL

ISSN 007/019X NLM Code B5Q

